# The direction of masked auditory category priming correlates with participants’ prime discrimination ability

**DOI:** 10.2478/v10053-008-0116-6

**Published:** 2012-08-21

**Authors:** Christina Bermeitinger, Dirk Wentura, Christopher Koppermann, Micha Hauser, Benjamin Grass, Christian Frings

**Affiliations:** 1 Department of Psychology, University of Hildesheim, Germany; 2 Department of Psychology, Saarland University, Saarbrücken, Germany; 3 Department of Psychology, University of Trier, Germany

**Keywords:** semantic priming, masked priming, auditory priming, semantic memory, negative semantic priming effect, category priming, auditory primes and targets

## Abstract

*Semantic priming* refers to the phenomenon that participants typically respond faster to targets following semantically related primes as compared to semantically unrelated primes. In contrast, Wentura and Frings (2005) found a negatively signed priming effect (i.e., faster responses to semantically unrelated as compared to semantically related targets) when they used (a) a special masking technique for the primes and (b) categorically related prime-target-pairs (e.g., fruit-apple). The negatively signed priming effect was most pronounced for participants with random prime discrimination performance, whereas participants with high prime discrimination performance showed a positive effect. In the present study we analyzed the after-effects of masked category primes in audition. A comparable pattern of results as in the visual modality emerged: The poorer the individual prime discrimination, the more negative is the semantic priming effect. This result is interpreted as evidence for a common mechanism causing the semantic priming effect in vision as well as in audition instead of a perceptual mechanism only working in the visual domain.

## Introduction

The semantic priming paradigm constitutes a traditional tool in cognitive psychology for studying the structure and processes of semantic memory. In this paradigm, usually a prime stimulus (e.g., a word) is presented before a target stimulus (typically a word as well), and participants have to respond only to the target (e.g., by naming it). Many studies have demonstrated that the response to a target word (e.g., *apple*) is facilitated when it is preceded by a related word (e.g., *fruit*), as compared to a condition in which it is preceded by an unrelated word (e.g., *bird*; for reviews, see Lucas, 2000; McNamara, 2005; Neely, 1991).

Usually, the *semantic priming effect* is explained in terms of automatic encoding facilitation (e.g., Anderson, 1983; see Masson, 1999, for a more recent approach), that is, a prime word facilitates the processing of a target word by automatically activating its representation even before the target word appears. However, there have been also attempts to explain semantic priming by strategic processes (e.g., Becker, 1980). For example, participants may generate expectations about which target words might follow a specific prime. If an expected word appears as the target, the response is facilitated. If semantic priming is due to strategic expectations, it would tell us only little about the processes and structure of semantic memory. Several variables can be manipulated as to hedge the semantic priming effect against alternative explanations in terms of strategies. For example, since the generation of expectancies is time-costly, the stimulus onset asynchrony (SOA) between the onset of the prime and the onset of the target can be made exceedingly short, thereby preventing the influence of controlled prime processing (e.g., Neely, 1977, 1991; Perea & Gotor, 1997).

The most straight-forward way to prevent controlled prime processing, however, is masking the prime, hereby preventing an explicit access to the prime’s meaning and preventing insight into the contingency of the stimulus sequences (i.e., that the target is often preceded by a related prime). The results from masked semantic priming studies are rather diverse (see e.g., Van den Bussche, Van den Noortgate, & Reynvoet, 2009): Some authors found evidence for priming effects with faster responses to related targets as compared to unrelated targets (e.g., Bodner & Masson, 2003), other studies revealed no priming effects with masked primes (e.g., Klinger, Burton, & Pitts, 2000), and there are studies which showed negative semantic priming effects, that is, faster responses to unrelated as compared to related targets (e.g., Carr & Dagenbach, 1990; Kahan, 2000).

However, comparing the results from masked and unmasked priming studies is difficult because the presentation times of the primes usually differ. In particular, masked primes are typically presented rather short (e.g., between 14 and 50 ms) whereas unmasked primes are typically presented rather long (e.g., between 100 and 300 ms). In turn, the differences in results between masked and unmasked primes can probably be explained by the differences in prime duration or prime energy (see also Dupoux, de Gardelle, & Kouider, 2008). To solve this confound between prime duration and presentation (i.e., masked vs. unmasked), Wentura and Frings (2005) introduced a new variant of masking by interchanging prime and mask rapidly and repeatedly. Thus, the summed prime duration of the masked prime was as long as that of an unmasked prime (in typical priming studies) albeit participants’ ability to access the meaning of the prime (as indexed by a test of participants’ prime discrimination performance after the experiment) was comparable to other masking studies. Using this masking technique with category labels as primes and category exemplars as targets, a negatively signed semantic priming effect (i.e., slower responses to related than unrelated targets) was found. This effect was especially present for words which were low dominant exemplars for their category (but see Bermeitinger, Frings, & Wentura, 2008; and Frings, Bermeitinger, & Wentura, 2008, who found no differences between low and high dominant exemplars) and for participants with low prime discrimination abilities according to a prime identification task following the priming task (Bermeitinger et al., 2008; Frings et al., 2008; Wentura & Frings, 2005).

Wentura and Frings (2005) suggested explaining the negative semantic priming effect from repeated masked primes in terms of the center-surround inhibition theory of Dagenbach and colleagues (e.g., Carr & Dagenbach, 1990; Dagenbach, Carr, & Barnhardt, 1990), but several other theories - for example, the retrospective prime clarification (RPC) theory (Kahan, 2000) or the ROUSE model (Huber, 2008; Huber & O’Reilly, 2003; Huber, Shiffrin, Lyle, & Quach, 2002; Huber, Shiffrin, Quach, & Lyle, 2002) - also can be related to the findings.^1^

Independently of the question which theory is better suited to explain the effects found with repeated masked primes, until now it is unclear whether the results of Wentura and Frings (2005) are limited to the particular masking technique with visual stimuli or whether they can be generalized supporting the hypothesis that a weakly activated concept - as due to the new priming technique with repeated masked primes - per se can lead to negative priming effects. Generally spoken, it is unclear whether the effect originates at perceptual or at semantic processing stages. In the present article, we used auditory stimuli for analyzing whether the effect found by Wentura and Frings is restricted to visually presented information or whether it is a general phenomenon that occurs independently of the primes’ modality. An auditory replication of the effects found by Wentura and Frings would argue for the assumption that the effect probably has to be located at semantic stages.

There is some debate on the similarities and differences of the neural architectures and processes underlying spoken and visual word recognition (e.g., Kouider & Dupoux, 2001). Yet, so far there are only a few semantic priming studies which used some kind of masking technique to present auditory material.^2^ For example, Kouider and Dupoux (2005) introduced a masking technique for auditory material by presenting time-compressed primes which are surrounded by time-compressed and time-reversed other words. Using associatively related or feature-overlapping prime-target pairs (e.g., *rabbit-carrot* or *cow-ox*, respectively), the authors found no evidence for semantic priming effects when prime audibility was low but positive priming effects for primes with a high prime audibility. At an abstract level, such a kind of presentation mimics the one realized with the repeated masked technique of Wentura and Frings (2005): Although time-compressed and therefore hard to identify, primes are presented rather long and with rather high intensity (i.e., they are presented in approximately standard sound level). Thus, it seems worthwhile to analyze what happens if the categorically related material as used by Wentura and Frings is presented auditorily and under masked conditions.

The present study had two aims. First, we implemented a long prime duration and a marginally perceptible prime presentation with a technique different from the repeated masked technique introduced by Wentura and Frings (2005). We hereby analyze whether the effects from repeated masked primes generalize to another presentation technique. In particular, we assume a correlation of the priming effect and the individual prime discrimination ability of participants. Participants with low prime discrimination should show a negatively signed priming effect whereas participants with high prime discrimination should show a positively signed priming effect. Second, by transferring our approach from the visual to the auditory modality, the experiment adds to the debate on whether perceived written and spoken speech rely on the same or on different neural architectures and processes (e.g., Kouider & Dupoux, 2001).

## Method

### Participants and design

The sample consisted of 67 students (47 female, 20 male) from the Saarland University. Their median age was 22 years (ranging from 19 to 41 years). All of them were native speakers of German and did not report any hearing deficit. They got partial course credits for their participation. The data of two further participants were discarded because their overall mean reaction time (RT) was above 900 ms.^3^

We used a two-factorial design. The first factor was priming condition (related, unrelated, neutral) which was varied within participants. In the neutral condition, we used time-reversed (i.e., meaningless) and time-compressed versions of words as primes. The neutral condition was only introduced in order to lower the overall rate of related prime-target pairs and was not further analyzed.

In addition and in accordance with other studies on categorical priming, dominance of the target exemplars (high- vs. low-dominance exemplar of the category) was varied within participants and orthogonally to the priming factor. Finally, target-lexicality (word vs. non-word) was varied within-participants to establish a meaningful task for participants. In accordance with other lexical decision studies, analyses were focused on word trials.

Furthermore, we measured the individual prime discrimination ability in a direct test of prime discrimination conducted after the main experiment. Data of this measure were used for correlation analyses.

### Material

Essentially, the visually presented material used by Wentura and Frings (2005) was adapted for auditory presentation. As in the experiments by Wentura and Frings, the prime set consisted of four labels of natural categories: *Frucht* (*fruit*), *Insekt* (*insect*), *Vogel* (*bird*), and *Blume* (*flower*). Three high-dominance and three low-dominance exemplars of each category served as target words. High-dominance exemplars had a mean association frequency (Mannhaupt, 1983) to their category label of 67.1% (*SD* = 10.7%; range 55% to 86.5%), whereas low-dominance exemplars had a mean association frequency of 6.2% (*SD* = 2.87%; range 2.5% to 11.5%). The average word frequency was 5.318 (*SD* = 10.026) for high-dominance exemplars, and 502 (*SD* = 727)for low-dominance exemplars (according to the German database of written language, COSMAS II). Mean length of the target words was 527 ms (ranging from 397 ms to 836 ms). For the lexical decision task, non-word targets were created by changing one phoneme of each target word (mean length of the non-word targets was 538 ms, ranging from 412 ms to 766 ms).

The prime and target set (see Table 1) was narrated by a professional male narrator and actor (the material was narrated in mono, sample format: 32 bit, sample frequency: 22050 Hz, maximum frequency: 8000 Hz). Thereafter, the auditory material was edited with the software Audacity. First, the original material was noise filtered and adjusted in sound level. Then, the primes were time-compressed to 25% of their original duration which resulted in a prime length of 264 ms for *Frucht* (*fruit*), 408 ms for *Insekt* (*insect*), 350 ms for *Vogel* (*bird*), and 321 ms for *Blume* (*flower*).

**Table 1.** Material Auditorily Presented as Primes (i.e., Categories) and Targets (Words, i.e., Category Exemplars, and Corresponding Nonwords)

*Related prime*	*Word target*	*Nonword target*
*Blume (flower)*	*Dahlie (dahlia)*	*Dahkie*
	*Krokus (crocus)*	*Krokes*
	*Lilie (lily)*	*Lulie*
	*Nelke (carnation)*	*Nelte*
	*Rose (rose)*	*Roze*
	*Tulpe (tulip)*	*Tolpe*
*Frucht (fruit)*	*Apfel (apple)*	*Apsel*
	*Banane (banana)*	*Banake*
	*Birne (pear)*	*Birno*
	*Dattel (date)*	*Dassel*
	*Feige (fig)*	*Feise*
	*Mango (mango)*	*Mange*
*Insekt (insect)*	*Biene (bee)*	*Biena*
	*Fliege (fly)*	*Fliepe*
	*Grille (cricket)*	*Grulle*
	*Motte (moth)*	*Motta*
	*Mücke (midge)*	*Müche*
	*Wanze (bedbug)*	*Wonze*
*Vogel (bird)*	*Amsel (blackbird)*	*Amtel*
	*Dohle (daw)*	*Dohlo*
	*Drossel (thrush)*	*Drissel*
	*Fasan (pheasant)*	*Fosan*
	*Schwan (swan)*	*Schwon*
	*Star (starling)*	*Ster*

**Figure 1.**
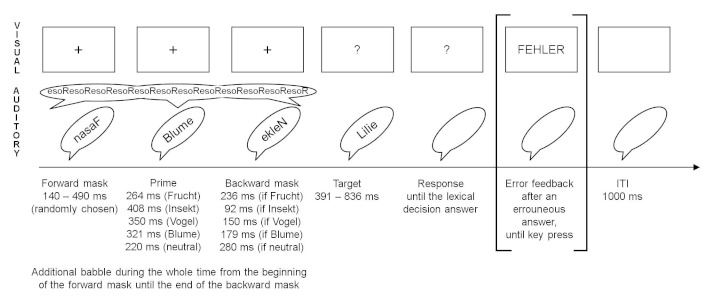
Visual and auditory procedure of a trial with examples for the forward mask (time-reversed and time-compressed presentation of *Fasan* = *pheasant*), the prime (time-compressed presentation of *Blume* = *flower*), the backward mask (time-reversed and time-compressed presentation of *Nelke* = *carnation*) with a duration which depends on the preceding prime (in the example, the duration of the backward mask is 179 ms as the prime was *Blume*; the stimulus onset asynchrony was held constant at 500 ms for all combinations of prime and backward mask), and an example for a target word (*Lilie* = *lily*); the corresponding target nonword would be *Lulie*. The example represents a related trial. For more details, see the Procedure section.

The neutral primes and the masks were created by time-reversing the time-compressed word and non-word targets. Neutral primes were shortened to 220 ms. The time-reversed and time-compressed target *Rose* (*rose*) was used as additional babble during the whole mask-prime-mask presentation (see Figure 1).

### Procedure

Participants were tested in groups of up to four persons at individual workstations. The experiment was conducted using the E-Prime software (version 1.1) with a standard PC, 17’’ CRT monitors (100 Hzrefresh rate), and Terratec HeadsetMaster 5.1 headsets. Viewing distance was about 60 cm. Instructions were given on the CRT screen. Participants were told that noise (comparable to noise in a station concourse) would be presented. Subsequent to the noise, a word or a non-word would be presented. Participants were requested to quickly and accurately categorize each word with regard to lexicality (by pressing the right/left key with their right/left index finger for correctly/incorrectly pronounced words, respectively). The sequence of each trial was as follows (see Figure 1): First, a randomly chosen forward mask was presented (length was between 140 ms and 490 ms). Then, the prime was presented. Subsequently, the randomly chosen backward mask was presented for 500 ms minus presentation time of the prime (thus, the mask was cut off after a time of 500 ms from the beginning of the prime; see Figure 1). Additionally, during this mask-prime-mask sequence, the additional babble (see Material section) was presented auditorily. On the screen, a fixation cross (+) was present during the whole mask-prime-mask sequence. Then, the target was presented. Target RT was measured from target onset. Primes and masks were 10% lower in intensity than the target, and the babble was 7% lower in intensity than the target (see Figure 1). The target was accompanied by a question mark on the screen. The question mark remained until the participant’s lexical decision answer. After an erroneous response, an error feedback appeared on the screen until participants pressed either the right or the left key. The intertrial interval (ITI) was 1,000 ms.

The experiment comprised three blocks with 48 trials each (16 related, 16 unrelated, and 16 neutral prime-target pairs; half of the trials with non-word targets). Over the course of the experiment, each target appeared once in each of the three priming conditions. Within a block, each target was presented in one of the three priming conditions. The sequence of priming conditions for a given target was determined by a Latin-square design (i.e., sequence of targets and conditions was balanced over participants). There was a short pause after every 24 trials. Before the experimental trials, there was a practice phase consisting of 48 trials with the same material (primes and targets) used in the main experiment. This practice block resembles the experimental Block 3 and was introduced to familiarize participants with the primes and targets used in the main part of the experiment. The procedural details of this practice block were adopted from Wentura and Frings (2005) and adapted to the auditory presentation. At the very beginning, there was a practice phase with 24 trials with targets from the categories trees and vegetables to familiarize participants with the headphones and the general procedure of the auditory presentation; in these trials, only neutral primes were presented. After the priming experiment, a direct test was conducted to test the individual prime discrimination ability. The trial procedure was the same as in the priming experiment with the following exceptions: first, no target was presented; second, participants had to decide via mouse click whether they heard either any word (button “word”) or no word (button “no word”) within the mask-prime-mask noise. When participants decided for “word”, they had to choose one of the four category labels or “another word” on the next display via mouse click. The direct test comprised 48 trials. Within these 48 trials, 32 trials were presented with one of the four category labels as primes (thus, each category label was presented eight times), the other 16 trials comprised neutral primes. Sequence of trials was randomly chosen. The direct test was practiced with six practice trials.

## Results

### Direct test

With *hits* defined as word-decisions if a word was presented and *false alarms* defined as the word-decisions if a non-word was presented, we calculated *d*’ as the canonical signal detection index. However, for *n* = 4 participants, *d*’ could not be calculated because these participants had a false alarm rate of zero. To account for this, we took two means. First, we followed the so-called *loglinear approach* (see Hautus, 1995; Stanislaw & Todorov, 1999) which involves adding 0.5 to both the number of hits and the number of false alarms and adding 1 to both the number of signal trials (i.e., word trials) and the number of noise trials (i.e., the non-word trials), before calculating the hit and false alarm rates. Mean *d*’ was 1.14 (*SD* = 0.49), a value that indicated moderate prime discriminability. Second, we calculated *A*’ as a nonparametric analogue to *d*’ (see Pollack, 1970; Pollack & Norman, 1964; see also Stanislaw & Todorov, 1999). *A*’ is defined to range from 0 to 1 with *A*’ = .5 indicating random responding. Mean *A*’ was .79 (*SD* = .09), a value that corresponds in interpretation to the mean of *d*’ (*d*’ and *A*’ correlated with *r* = .95).

Since we asked participants to indicate the word identity if they had responded with “word”, we additionally calculated Kappa (Cohen, 1960) for the concordance in the 5 (Stimulus: word 1 to 4; nonword) × 5 (Response: word 1 to 4; nonword) table.^4^ Mean Kappa was *K* = .46(*SD* = .15), again a value that indicated moderate prime discriminability. (*K* is defined to range from -1 to 1 with *K* = 0 indicating no concordance.)

### Priming effects

Mean RTs (see Table 2) were derived from correct responses to word targets. The mean error rate for these trials was 11.6%. RTs that were 1.5 interquartile ranges above the third quartile with respect to the individual distribution (Tukey, 1977), were above 1,500 ms, or were below 200 ms were discarded (3.3% of all trials with word targets). Preliminary analyses showed no significant differences with regard to the dominance factor, neither in analyzing overall priming nor with regard to the correlational analyses. Therefore, we discarded the factor for the sake of brevity (for a discussion of this factor, see also Frings et al., 2011).

**Table 2.** Mean Reaction Times and Mean Error Rates of Word and Nonword Targets as a Function of Priming Condition (Related, Unrelated, Neutral), and Quintile (According to *d*’, See Text).

	*d*’	Related		Unrelated		Neutral	
		RTs	Error rates	RTs	Error rates	RTs	Error rates
Word targets							
Overall	1,14	705 (62,3)	11,2 (7,2)	711 (67,7)	12,1 (7,9)	710 (59,7)	11,4 (7,2)
Quintile 1	0,45	705 (62,8)	13,1 (7,0)	703 (64,1)	13,8 (8,2)	706 (62,8)	12,5 (5,6)
Quintile 2	0,83	725 (58,0)	11,2 (9,1)	702 (58,1)	15,1 (7,7)	714 (57,0)	13,1 (7,4)
Quintile 3	1,10	708 (53,5)	9,9 (6,3)	724 (60,3)	9,0 (6,3)	725 (46,7)	10,3 (7,1)
Quintile 4	1,40	712 (78,6)	10,0 (5,2)	732 (88,6)	9,4 (7,0)	715 (78,6)	9,4 (7,5)
Quintile 5	1,85	672 (48,0)	11,9 (8,7)	690 (60,1)	13,8 (9,4)	690 (48,6)	12,2 (8,4)
Nonwords targets							
Overall	1,14	776 (70,8)	8,6 (5,7)	776 (77,8)	9,5 (5,6)	772 (70,6)	8,6 (6,4)
Quintile 1	0,45	768 (75,8)	9,3 (5,9)	764 (79,2)	7,4 (4,5)	770 (69,8)	9,0 (7,0)
Quintile 2	0,83	788 (68,8)	8,3 (4,8)	792 (92,3)	10,6 (6,5)	788 (102,1)	7,4 (7,2)
Quintile 3	1,10	794 (48,0)	9,0 (5,9)	798 (49,1)	8,0 (4,3)	786 (38,1)	10,9 (8,3)
Quintile 4	1,40	791 (92,2)	10,0 (6,1)	793 (90,4)	8,3 (6,7)	781 (68,7)	9,4 (5,1)
Quintile 5	1,85	738 (48,8)	6,4 (5,8)	730 (52,2)	8,7 (5,8)	731 (52,8)	8,0 (4,3)

*Note*. Standard deviations in parentheses.

**Figure 2.**
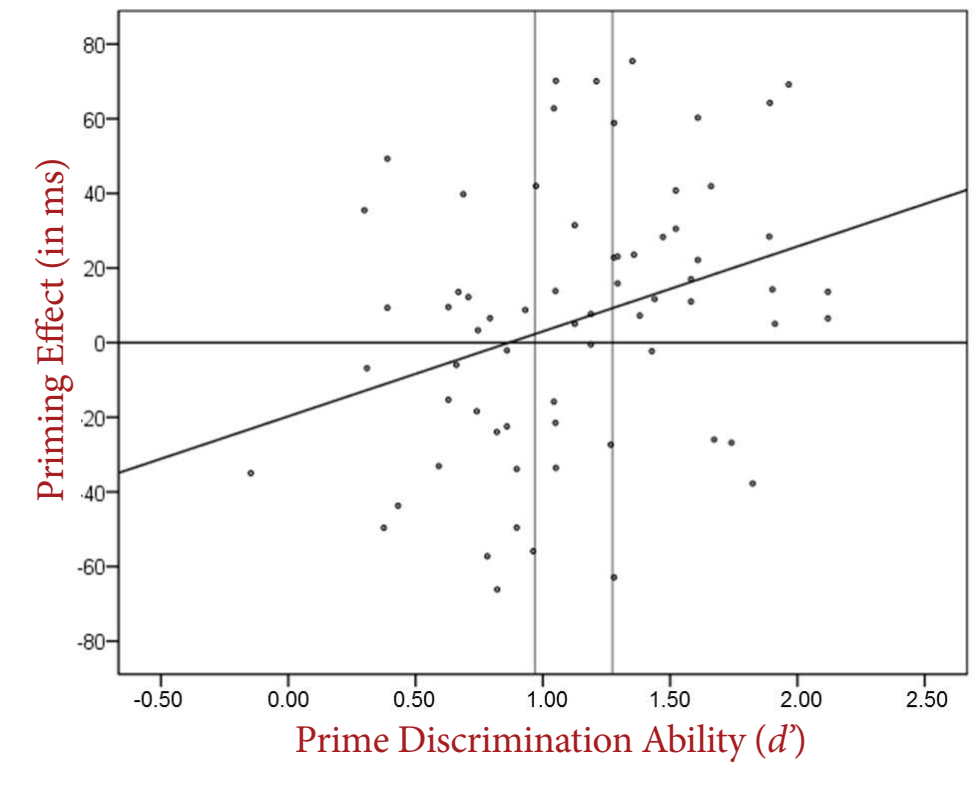
Scatterplot of priming on *d*’. The area within the vertical lines highlights Quintile 3 which was excluded from the main quintile analysis.

First, we computed priming effects as the difference in mean RT between related and unrelated prime-target pairs. Overall there was a priming effect of *M* = 6 ms (*SE* = 4). That is, numerically mean RT in related trials was faster than mean RT in unrelated trials. The effect however, failed to be significantly different from zero, *t*(66) = 1.41, *p* = .17. Most important for our rationale, however, is the fact that the overall priming effect significantly correlated with *d*’, *r*(65) = .32, *p* = .01; *A*’: *r*(65) = .30, *p* = .01; and with *K*, *r*(65) = .27, *p* = .03. That is, the better the individual prime discrimination, the larger and more positive is the priming effect. Figure 2 depicts the scatterplot of priming on *d*’.

We further explored the two extreme groups with low and high *d*’ values leaving out the third quintile (i.e., the area within the vertical lines in Figure 2). The subsample with low *d*’ values (i.e., Quintiles 1 and 2) is associated with a reversed priming effect, *M* = -13 ms (*SE* = 6 ms), *t*(25) = 2.08, *p* = .048,^5^ whereas the subsample with high *d*’ values (i.e., Quintiles 4 and 5) is associated with a positive priming effect, *M* = 19 ms (*SE* = 6 ms), *t*(27) = 3.17, *p* = .004.^6^

For errors, there was neither a significant priming effect, *M* = 0.93 (*SE* = 0.99), *t*(66) < 1, nor a significant correlation with *d*’, *A*’, or *K*, *r*(65) = -.05, *p* = .71, *r*(65) = -.05, *p* = .66, and *r*(65) = .11, *p* = .37, respectively.

## Discussion

We conducted an auditory semantic priming study with marginally perceptible category primes and clearly perceptible category exemplars as targets. We found clear evidence for a moderation of semantic priming by the prime discrimination ability of participants, that is, the individual prime discrimination ability correlated significantly with the priming effect. For participants with high performance in the prime discrimination test, which was measured in a direct test of prime discrimination ability following the main experiment, we found a positive priming effect, that is, presenting the corresponding category label facilitates processing of the target exemplar. For participants with low performance in the prime discrimination test, however, we found a negative effect, that is, presenting the corresponding category label impedes processing of the target exemplar.

The pattern of results extends the effects found by Wentura and Frings (2005; see also Bermeitinger et al., 2008; Frings et al., 2008) from the visual to the auditory modality. The negative semantic priming effect originally found with repeated masked category primes in the visual domain can be found even within another domain and with another kind of masking. Thus, the effect does not hinge on the special masking technique or perceptual mechanisms solely working when repeatedly masking visual words. The replication suggests that the mechanism responsible for the negatively signed priming effect occurs at a semantic representation level - instead of a perceptual representation level - or, at least, that the same (perceptual) mechanism works on the visual and the auditory modality. Thus, the results presented here make a strong case for the generalization of the data pattern found by Wentura and Frings (2005).

The original experiments of Wentura and Frings (2005) showed negative priming effects especially for low dominant target exemplars. This finding was not replicated by several subsequent studies (e.g., Bermeitinger et al., 2008; Frings et al., 2008) or by the current experiment. Yet, Wentura and Frings already argued that there is some arbitrariness in the classification of words into high dominant and low dominant exemplars. Most likely, for some participants a lot of exemplars might be comprised central to a category whereas for other participants only some highly prototypical exemplars might constitute a category and other exemplars may be only loosely or not related to the category. It was not the purpose of the present experiment to investigate individual differences in the representation of categories or to contribute to the question which words exactly represent the center of a category (for an experimental manipulation of target’s dominance, see Frings et al., 2011). Thus, also for the present experiment one has to assume that individual differences in the representation of categories precluded differences between priming effects for high and low dominant targets.

The current experiment and studies using the repeated masking technique in the visual domain (e.g., Bermeitinger et al., 2008; Frings et al., 2008; Wentura & Frings, 2005) revealed rather good performance in the direct tests. Thus, we had to discuss the results in relation to the issue of participants’ prime discrimination ability. First, good performance in the direct test after the priming experiment cannot be interpreted as evidence that participants also had more knowledge of the presence or the semantic content of the primes during the priming part of the experiment in which they had no previous knowledge about the presence of words within the stream of noise and in which they were not instructed to attend to other words than target words. However, we could assume that participants with low prime discrimination performances in the direct test also had no knowledge regarding the primes during the priming part. For these participants, it could be taken for granted that they did not perceive the primes at a conscious level. In conclusion, the results of the direct test do not reflect directly the degree of conscious prime perception during the preceding priming experiment. Second, there is a long going debate in cognitive psychology whether behavior can be influenced by stimuli that are presented subliminally. In particular, the criteria for “truly subliminal perception” were discussed (e.g., Forster, Mohan, & Hector, 2003; Holender, 1986; Merikle, Smilek, & Eastwood, 2001), and there are different recommendations how to detect unconscious cognition (e.g., Schmidt, 2007). With respect to this debate, repeated masked priming as well as the presented masking technique for auditory material would clearly not be considered as a truly subliminal presentation. Yet, the absolute level of prime discrimination abilities is not of large interest for this line of research as we found *qualitative* differences (i.e., negative instead of positive priming effects) in visual and auditory priming for participants with a low discrimination performance. It must be acknowledged, however, that it remains open for future research to identify possible cognitive processes - beyond differences in the performance of the direct test - which might lead to either negative or positive priming effects.

As outlined in the Introduction section, there are only few studies using marginally perceptible primes in the auditory modality. Our results confirm that semantic priming effects using marginally perceptible auditory primes can be observed. In addition, our results suggest that semantic effects in audition mimic those found in vision. This is especially interesting against the background of the debate whether words have only visual-specific versus auditory-specific representations or also more abstract representations which are accessible by the auditory and visual processing systems (e.g., Gipson, 1986; Kouider & Dupoux, 2001). The parallel results from marginally perceptible category primes in audition and vision suggest the conclusion that we also deal with an abstract representation (i.e., a “pure” semantic representation) of concepts or at least that auditory and visual stimuli can activate the same features which constitute the representation of concepts and which are responsible for priming effects.

Taken altogether, we demonstrated here that the negatively signed semantic priming effect - originally found with a repeated masking technique in the visual domain - can be replicated with auditory stimuli. This result is interpreted as evidence for a common semantic representation of concepts and a mechanism that is independent of the originally repeated masking method introduced by Wentura and Frings (2005).

## Footnotes

1 It should be noted that Avons et al. (2009) recently found no priming with the repeated masking technique for associatively related prime-target pairs. The restriction to categorically related stimuli fits an explanation in terms of intra-categorical center-surround inhibition processes.

2 There are also some articles on the question of cross-modal semantic priming, for example, using marginally perceptible auditory presented primes and visual words and vice versa (e.g., Lamy, Mudrik, & Deouell, 2008). However, these articles and the debate, for example, on cross-modal integration are only of low interest for the following experiment.

3 Including these two participants does not essentially change the results (see Footnote 5 for one slight difference).

4 Calculation of Kappa is based on the entries of the main diagonal of the 5 × 5 matrix (observed concordance) and the row and column margins (that are used to calculate the expected frequencies of the concordance cells). Responses of the type “another word” (which by definition had no concordance on the stimulus side) were added to the response margins of the word 1 to 4 response types (e.g., if words 1 to 4 were chosen as a response in 8, 7, 9, and 6 trials, respectively, and “another word” was chosen as a response in four trials, we used 9, 8, 10, and 7 as the word 1 to 4 marginal counts for this participant).

5 If the participants who were excluded because of their overall very high response times (> 900 ms, see Participants section and Footnote 3) were included into the analysis, the quintiles were built slightly differently. In consequence, the reversed priming effect for Quintiles 1 and 2 would be significant only in a one-tailed test.

6 Combining Quintiles 3, 4, and 5 yields a significant positive effect as well, *M* = 18 ms (*SE* = 5 ms), *t*(40) = 3.49, *p* = .001.

## Author note

The research reported in this article was supported by a grant of the Deutsche Forschungsgemeinschaft to Dirk Wentura and Christian Frings (WE 2284/5-2). Thanks are offered to Ingrid Scharlau, Michael Masson, Daniel Voyer, Glen Bodner, and four anonymous reviewers for providing helpful comments. Many sincere thanks go to Freddy Neumann for narrating the auditory material.
